# Askin Tumor: CT and FDG-PET/CT Imaging Findings and Follow-Up

**DOI:** 10.1097/MD.0000000000000042

**Published:** 2014-06-04

**Authors:** Tingting Xia, Yubao Guan, Yongxin Chen, Jingxu Li

**Affiliations:** Department of Radiology (TX, YG, JL), The First Affiliated Hospital of GuangZhou Medical University, Guangzhou, 510120; Department of Radiology (YC), State Key Laboratory of Oncology in South China, Cancer Center, Sun Yat-sen University, Guangzhou, 510060, PR China.

## Abstract

The aim of the study was to describe the imaging findings of Askin tumors on computed tomography (CT) and fluorine 18 fluorodeoxyglucose-positron emission tomography (FDG-PET/CT).

Seventeen cases of Askin tumors confirmed by histopathology were retrospectively analyzed in terms of CT (17 cases) and FDG-PET/CT data (6 cases).

Fifteen of the tumors were located in the chest wall and the other 2 were in the anterior middle mediastinum. Of the 15 chest wall cases, 13 demonstrated irregular, heterogeneous soft tissue masses with cystic degeneration and necrosis, and 2 demonstrated homogeneous soft tissue masses on unenhanced CT scans. Two mediastinal tumors demonstrated the irregular, heterogeneous soft tissue masses. Calcifications were found in 2 tumors. The tumors demonstrated heterogeneously enhancement in 16 cases and homogeneous enhancement in 1 case on contrast-enhanced scans. FDG-PET/CT images revealed increased metabolic activity in all 6 cases undergone FDG-PET/CT scan, and the lesion SUVmax ranged from 4.0 to 18.6. At initial diagnosis, CT and FDG-PET/CT scans revealed rib destruction in 9 cases, pleural effusion in 9 cases, and lung metastasis in 1 case. At follow-up, 12 cases showed recurrence and/or metastases, 4 cases showed improvement or remained stable, and 1 was lost to follow-up.

In summary, CT and FDG-PET/CT images of Askin tumors showed heterogeneous soft tissue masses in the chest wall and the mediastinum, accompanied by rib destruction, pleural effusion, and increased FDG uptake. CT and FDG-PET/CT imaging play important roles in the diagnosis and follow-up of patients with Askin tumors.

## INTRODUCTION

Primitive neuroectodermal tumor (PNET) is a rare type of malignant small round cell tumor, which can be further differentiated into central PNET and peripheral PNET (pPNET). Peripheral PNETs arising in the thoracopulmonary region have been termed Askin tumors and usually develop in the soft tissues of the chest wall and, occasionally, in the mediastinum or the periphery of the lung.^1–4^ There are few reports regarding the computed tomography (CT) and magnetic resonance imaging (MRI) findings of Askin tumors,^5–9^ and very few reports (1 case report) discussing fluorine 18 (18 F) fluorodeoxyglucose-positron emission tomography (FDG-PET)/CT imaging findings.^10–12^ Here, we present the CT (17 cases) and FDG-PET/CT (6 cases) imaging findings and follow-up of 17 cases of Askin tumors originating in the chest wall and mediastinum to improve our knowledge base regarding this malignant neoplasm.

## MATERIALS AND METHODS

### Study Subjects

From our hospital database, we identified patients who had received treatment at our hospital between January 2006 and December 2012. In total, 17 patients with chest wall and mediastinal Askin tumors were included in this study. For this retrospective study, no review board approval was required.

### CT Examination

All 17 patients had undergone unenhanced and contrast-enhanced CT scans, and 6 patients had also undergone FDG-PET/CT imaging. CT imaging was performed using Multi-Slice CT (Aquilion 16 and Aquilion 4, Toshiba, Yokohama, Japan), with a tube potential of 135 kV, a current of 350 mA, field of view of 250 mm × 250 mm, and reconstruction slice thickness of 2 mm and 7 mm. The contrast agent iohexol was used at a concentration of 300 mg I/mL. The dose used was 1.5 mL/kg, with an injection flow rate of 1.5 mL/s to 2.5 mL/s. CT scans were obtained 60 s after administering the contrast agent.

### FDG-PET/CT Acquisition

FDG-PET/CT imaging was performed using a dedicated PET/CT system (Discovery ST-16; General Electric Medical System, Milwaukee, WI). The patients were instructed to fast for 6 hours prior to 18F-FDG injection. Blood glucose was measured before the injection of the tracer to ensure that the glucose levels were below 8.1 mmol/L. We injected 18F-FDG (4.4–7.4 MBq/kg) intravenously and asked the patients to lie comfortably in a dark room for 45 min to 60 min. Patients were scanned from calvarium to mid-femur while in the supine position. The CT scan was performed prior to the PET scan, and the resulting data were used to generate a PET attenuation correction map. PET images were reconstructed with a slice thickness of 3.75 mm using the iterative ordered-subsets expectation maximization method. PET, CT, and fused PET/CT images were generated for review on a Xeleris computer workstation.

Two experienced radiologists reviewed each lesion on the CT and FDG-PET/CT images for tumor number, distribution, shape, attenuation, and other associated findings. For FDG-PET/CT imaging analysis, the maximal standardized uptake value (SUVmax) of FDG uptake was recorded for each lesion.

## RESULTS

### Clinical Data

The study group consisted of 9 males and 8 females with a mean age of 15 years (range 4–39 years) between January 2006 and December 2012. Fourteen patients presented with a chest wall mass, 8 presented with cough, and 5 presented with shortness of breath and local chest pain.

### CT Findings

**FIGURE 1:**
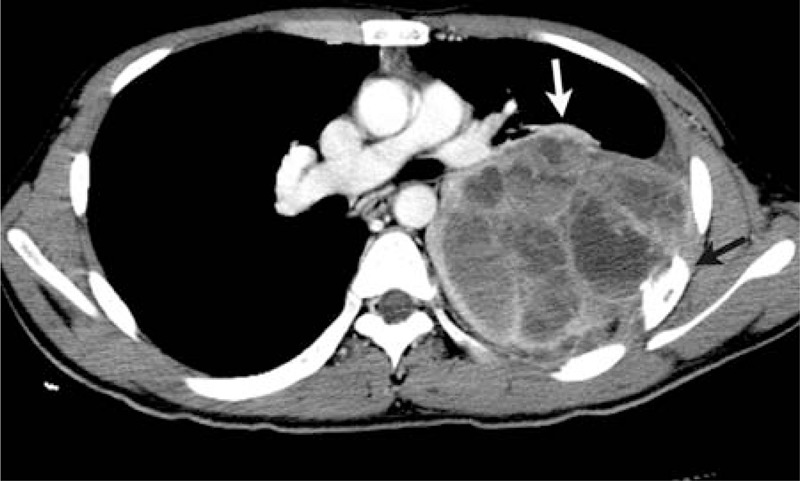
A 16-year-old man complained of left-side thoracic pain and shortness of breath for about 1 month. The tumor showed the heterogeneous enhancement in the posterior left chest wall (white arrow) and destruction of the rib adjacent to the tumor (black arrow).

**FIGURE 2:**
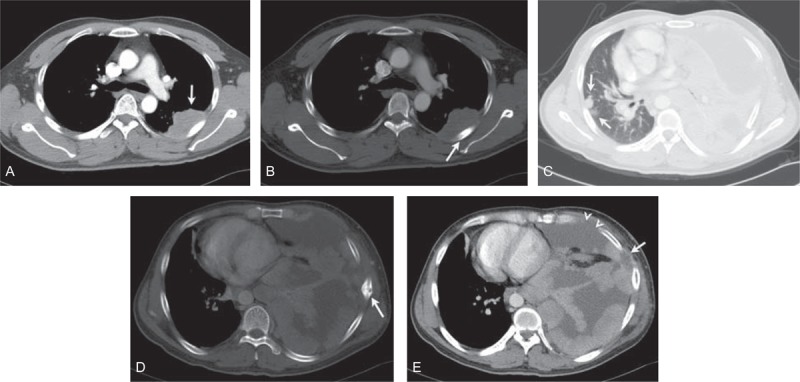
A 14-year-old man presented with a history of left chest pain about 1 month and slight swelling on the left chest wall. The tumor showed the homogeneous enhancement in the posterior left chest wall (A, arrow); the rib adjoining the tumor showed bony destruction in the bone window (B, arrow). The tumor showed right lung metastasis (C, arrow), left rib metastasis (D, arrow) and left pleural metastasis (E, arrow), and pleural effusion (E, arrowhead) at the 6-month follow-up after surgery and chemotherapy.

**FIGURE 3:**
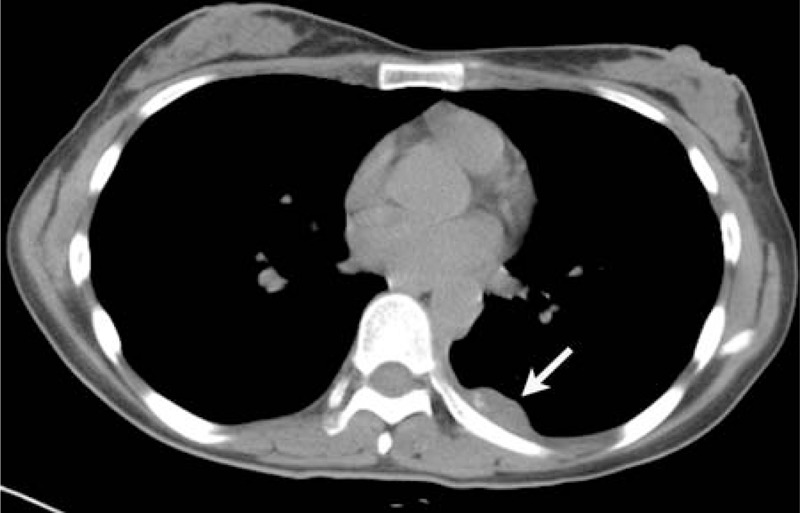
A 22-year-old woman presented with a history of left back pain for about 1 month. Unenhanced CT scan shows that the tumor had the amorphous calcifications in the posterior left chest wall (arrow).

**FIGURE 4:**
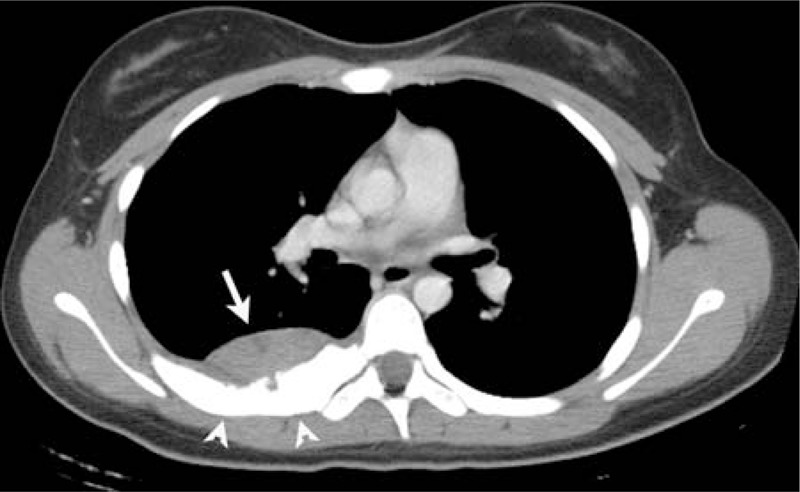
A 19-year-old woman presented with a history of right chest pain for about 1 month. Enhanced CT scan shows that the tumor had mild heterogeneous enhancement in the posterior right chest wall (arrow) and bony destruction (arrowhead).

Eight chest wall Askin tumors occurred on the left side and 7 occurred on the right side. CT scans showed irregular chest wall soft tissue masses. The largest diameter of the soft tissue tumors ranged from 5.1 cm to 13.7 cm. Thirteen cases demonstrated heterogeneous masses, of which 9 contained cystic components and necrotic tissue, and 2 cases demonstrated homogeneous masses on unenhanced CT scan (Figures 1 and 2). Calcifications were found in 2 cases, and 9 cases exhibited rib destruction on unenhanced CT scans (Figure 3). The tumors showed homogeneous mild enhancement in 1 case, heterogeneous mild enhancement in 5 cases, and heterogeneous moderate enhancement in 9 cases on contrast-enhanced scans (Figure 4). Pleural effusion was found in 7 cases (4 were small, 2 were moderate, 1 was large).

**FIGURE 5:**
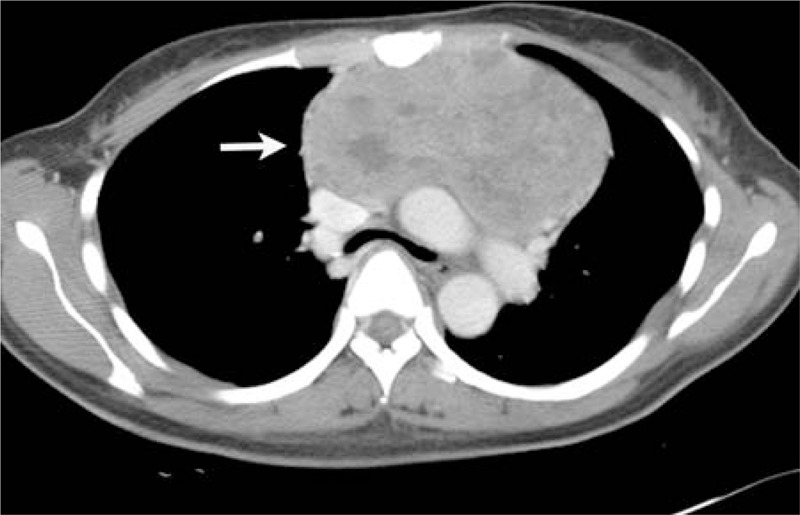
A 17-year-old man presented with a history of cough for about 2 months. Enhanced CT scan shows that the anterior middle mediastinal tumor had mild-to-intermediate heterogeneous enhancement, and adjacent large vessels were encased and displaced (arrow).

Two mediastinal Askin tumors arising in the anterior middle mediastinum showed heterogeneous masses on unenhanced CT and heterogeneous moderate enhancement with cystic components and necrosis on contrast-enhanced CT scans. The tumors invaded the anterior chest wall and encased mediastinal vessels (Figure 5). Small pleural effusions were seen in 2 cases.

### FDG-PET/CT Imaging Findings

**FIGURE 6:**
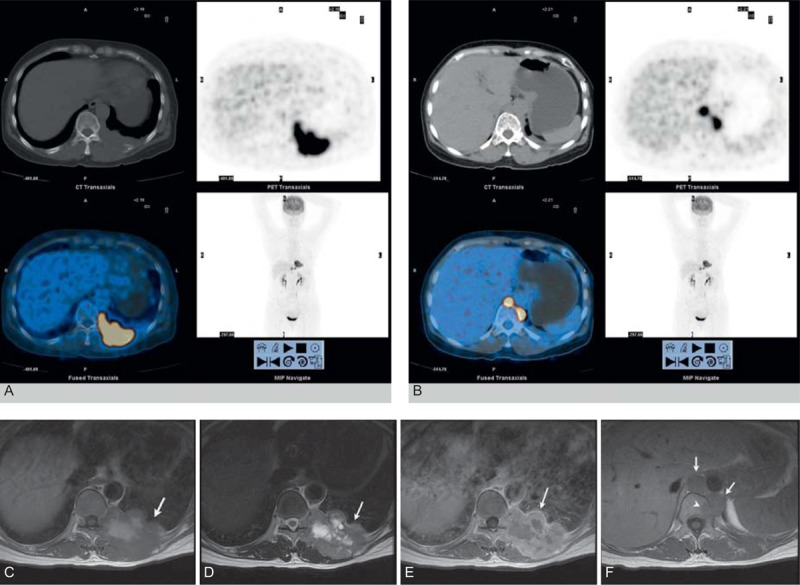
A 20-year-old man presented with a history of left back pain for about 1 month and fever for 5 days. FDG-PET/CT images (A, B) show increased metabolic activity in the left chest wall mass, the retrocrural lymph nodes, and bony destruction. The current SUVmax of this lesion was 18.6. The T1-weighted image (C) and the T2-weighted image (D) show that the tumor had heterogeneous intermediate and high signal intensity (arrow). The enhanced T1-weighted image (E) shows that the tumor had intermediate heterogeneous enhancement (arrow). In addition, at the 4-month follow-up after surgery and chemotherapy, the T1-weighted image (F) shows that retrocrural lymph nodes were enlarged (arrow) and the bodies of thoracic vertebrae were destroyed (arrowhead).

**FIGURE 7:**
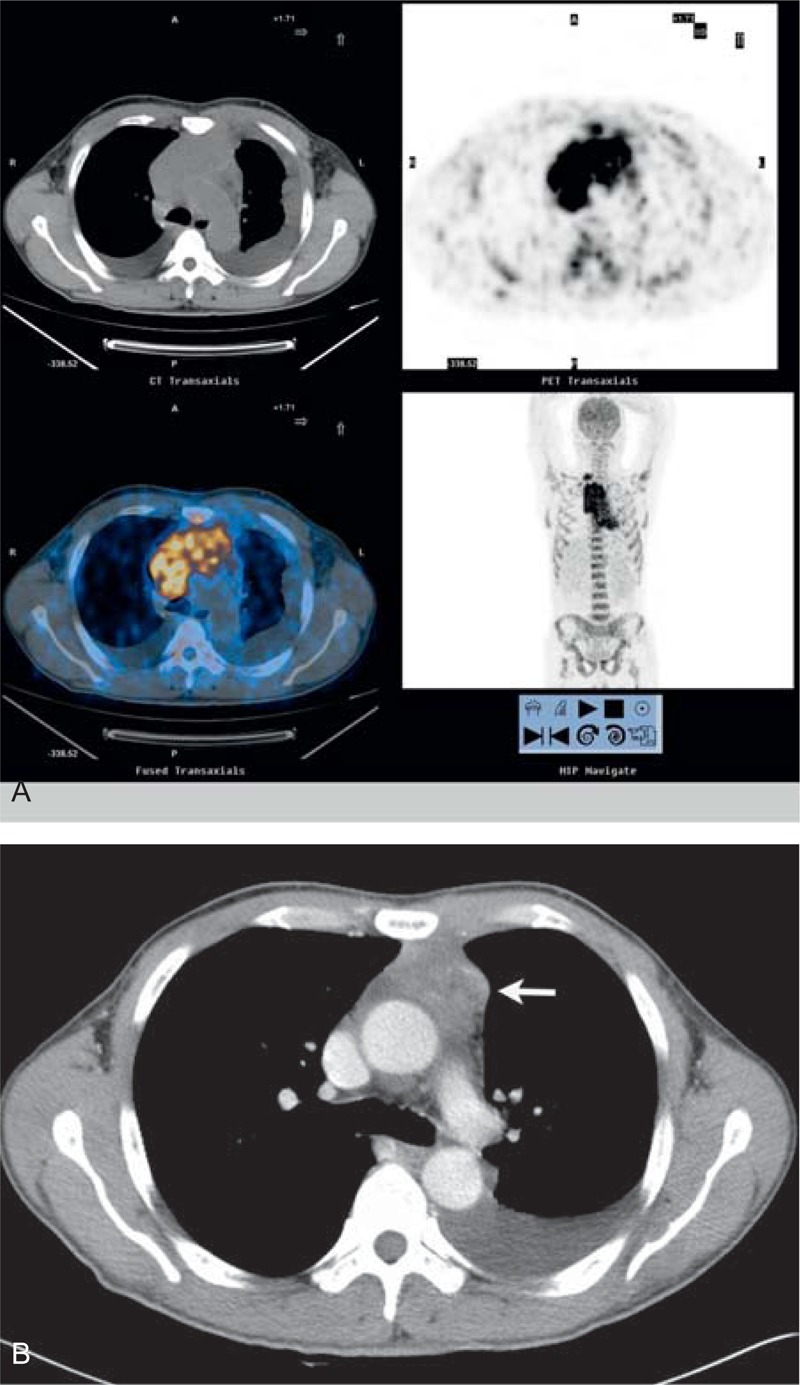
A 23-year-old man presented with a history of chest pain and shortness of breath for about 1 month. FDG-PET/CT images (A) show increased metabolic activity in the anterior middle mediastinal tumor, lymph nodes in the right supraclavicular fossa, and small bilateral pleural effusions; CT scan (B) shows that the tumor was remission at the 3-month follow-up after chemotherapy (arrow).

FDG-PET/CT images revealed increased metabolic activity in the chest wall and mediastinal masses in all 6 cases, in agreement with the CT images. The lesion SUVmax ranged from 4.0 to 18.6. One patient with a posterior left chest wall tumor also had increased metabolic activity in the retrocrural lymph nodes (Figure 6). One patient with a mediastinal tumor also had increased metabolic activity in a lymph node in the right supraclavicular fossa and bones (Figure 7). No foci of increased metabolic activity were observed in lymph nodes or other organs in the other 4 patients who had undergone FDG-PET/CT imaging.

### Microscopy and Immunohistochemistry

**FIGURE 8:**
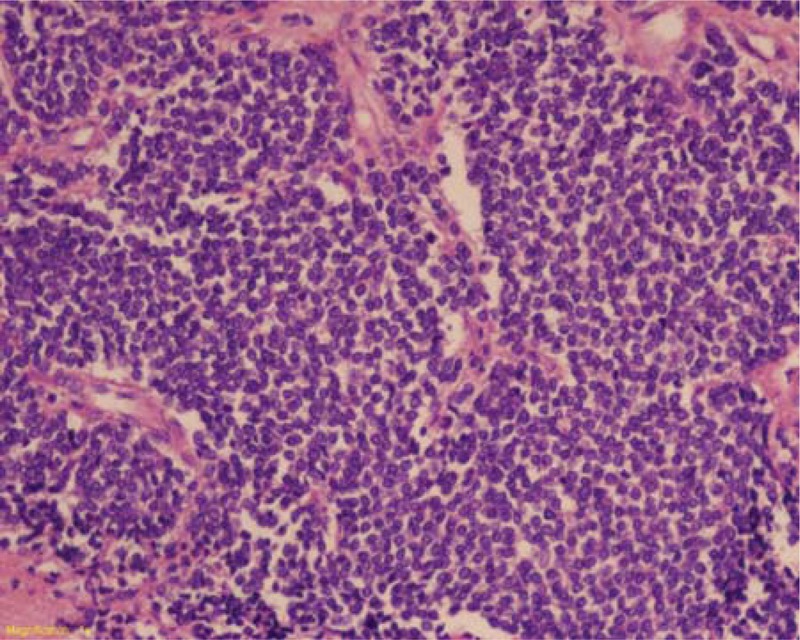
Open surgical biopsy demonstrated the pathological features of Askin tumors (HE × 200). Askin tumors were composed of sheets of small round cells with tinged cytoplasm and frequent Homer-Wright rosettes.

The cut surfaces were yellowish white or grayish white in color. Patchy cystic areas were observed in 10 cases and patchy hemorrhage was seen in 1 case. Histological examination showed that the tumors consisted of medium round or short fusiform-shaped cells with little cytoplasm. Many tumor cells showed increased mitotic counts and cellular necrosis. Homer-Wright rosettes were observed in 10 cases (Figure 8). Immunohistochemical studies revealed that 15 tumors were positive for CD99, 13 for neuron-specific enolase (NSE), 12 for vimentin, and 10 for S100-protein.

### Treatment and Follow-Up

Three and 10 patients with chest wall Askin tumors underwent surgical resection before and after chemotherapy, respectively. The other 4 patients underwent chemotherapy.

Four of the 13 patients with chest wall tumors who had undergone surgery died of lung and bone metastases, and 7 patients developed lung and liver metastases and/or recurrence at the 2 to 6-year follow-up. One patient showed no tumor recurrence or metastasis, and 1 patient was lost to follow-up.

Two patients with chest wall tumors with distant metastases demonstrated partial relapse at the 6-month to 1-year follow-up after chemotherapy. Of the 2 patients with mediastinal tumors who had been treated with chemotherapy, 1 suffered a partial relapse at 18 months and the other developed multiple bone metastases.

## DISCUSSION

In 1979, Askin et al^1^ first described a rare, malignant small, round cell tumor that originated from primitive undifferentiated neuroepithelial cells. This type of tumor consists of small round cells that are densely arranged, and Homer-Wright rosettes are usually seen around the fibrin matrix. The tumor cells showed active endomitosis, little cytoplasm, nuclear condensation, and a high nucleus–cytoplasm ratio.

Askin tumors are a type of pPNET. Histopathological studies show that pPNET and Ewing’s sarcoma (ES) result from a reciprocal translocation of the long arms of chromosomes 11 and 22, t (11; 22)(q24;q12), and are at different stages of neurodifferentiation. Both tumor types are grouped as the ES/PNET family and are difficult to distinguish from each other based on histomorphology. Neuronal differentiation features observed with electron microscopy and immunohistochemistry can help differentiate between the 2 types of tumor. The pPNET tumor cells reveal neuronal differentiated structures, such as microfilaments, microtubes, dense core granules, and rare synapses, under electron microscopy. Immunohistochemically, the pPNET tumor cells contain specific neural differentiation markers, such as CD99, NSE, vimentin, CgA, and S100-protein.^13,14^ In our series, at least 2 neuronal markers were expressed in each case.

Askin tumors are predominantly seen in children and teenagers and are more common in females than in males.^1^ In our series, 11 patients were children and teenagers while only 2 were over the age of 30. These ages are similar to previous reports, but gender differences were not found in our series.

Askin tumors usually originate from the chest wall and often demonstrate heterogeneous soft tissue masses with cystic lesions and necrotic areas as well as rib invasion, destruction, and pleural effusions.^5–9^ In our series, 13 chest wall tumors demonstrated heterogeneous masses, of which 9 showed cystic degeneration and necrosis, and 2 small tumors consisted of homogeneous masses, which is consistent with previous studies.^5–9^

Askin tumors of the mediastinum are rare,^15–17^ and only 1 previous report^17^ has focused on mediastinal Askin tumors. Two mediastinal Askin tumors in our series demonstrated heterogeneous soft tissue masses, invaded the anterior chest wall, and pushed and encompassed the neighboring vessels. The tumors showed imaging findings similar to previously reported results.^17^

Pleural effusions, caused by pleural invasion, are another important finding associated with Askin tumors. In our series, pleural effusions were found in 7 chest wall Askin tumors (4 were small, 2 were moderate, and one was large). Two mediastinal Askin tumors showed small or moderate-sized pleural effusion because of pleural invasion.

Calcifications are a rare finding in Askin tumors. Fink et al^5^ reported calcifications in 1 out of 10 Askin tumors, while Sabate et al^8^ found calcifications in 2 out of 8 Askin tumors. In our series, calcifications were observed in 2 chest wall Askin tumors.

At initial diagnosis, remote metastases are uncommon in Askin tumors of the chest wall. Winer-Muram et al^6^ reported mediastinal lymph node metastases in 2 out of 8 Askin tumors, while Sabate et al^8^ reported mediastinal lymph node metastases in 1 out of 8 Askin tumors. In our series, bilateral lung metastases were found in 1 chest wall Askin tumor with no signs of mediastinal lymph node metastases.

There are few case reports concerning 18F-FDG PET/CT findings of Askin tumor.^10–12^ Musana et al^18^ did not report any increase in FDG uptake in the large right forearm soft tumor of a 36-year-old female with pPNET. However, Kara Gedik et al^10^ reported a chest wall Askin tumor with a 4.36 SUVmax in a 73-year-old patient, and no additional findings regarding lymph node and distant metastases were noted. Demir et al^11^ and Kamaleshwaran et al^12^ also reported the focal FDG uptake in 2 males (aged 17 years and 18 years) with Askin tumors with a SUVmax of 8.3. All 3 reports concerning chest Askin tumors contradicted the report by Musana et al.^18^ In our series, both chest wall and mediastinal Askin tumors revealed increased metabolic activity in all 6 cases that had undergone FDG-PET/CT scanning with the lesion SUVmax ranging from 4.0 to 18.6. We speculated that the differences in uptake between Askin tumors and right upper extremity PNET might be attributed to differences in biological behavior and metabolic features at the different onset sites.

Local recurrence and remote metastases are common in Askin tumors after treatment. The most common findings of recurrence are local chest wall masses, intrapulmonary metastases, and enlarged mediastinum lymph nodes, as well as metastasis to the bone, liver, adrenal glands, or the retroperitoneal space.^9^ In our series, 12 patients suffered recurrence and/or lung or bone metastases after surgery or chemotherapy, and 1 was lost to follow-up after chemotherapy. Four patients showed improvement or remained stable after treatment at the 6 to 18-month follow-up.

The patient series is heterogeneous in the study; all patients were evaluated by CT but only 6 cases by FDG-PET/CT. More studies and cases of FDG-PET/CT are needed for drawing the conclusion that FDG-PET/CT method precedes CT method for diagnosis of Askin tumor.

Askin tumor needs to be differentiated from chest mass occurring in children and adolescents, including ES, rhabdomyosarcoma, neuroblastoma, and ganglioneuroblastoma. ES originated from rib and rhabdomyosarcoma can often present a soft tissue mass occurring in chest wall, with bone destruction and pleural effusion. These tumors show similar imaging features to Askin tumor and are very difficult to identify preoperatively. Neuroblastoma and ganglioneuroblastoma are evidently enhanced early and may invade adjacent tissues and blood vessels. They may have osseous metastasis with coarse calcification in the lesions.^19^

In conclusion, CT and FDG-PET/CT images of Askin tumors showed heterogeneous soft tissue masses in the chest wall and the mediastinum accompanied by rib destruction, pleural effusions, and increased FDG uptake. Therefore, Askin tumor should be taken into account in the differential diagnosis when a chest wall mass is identified in children and young adults. CT and FDG-PET/CT imaging can also play important roles in follow-up of patients with Askin tumors.
